# A Rare Case of Ectopic Hidradenoma Papilliferum of the External Auditory Canal

**DOI:** 10.7759/cureus.61237

**Published:** 2024-05-28

**Authors:** Stephanie V Shimon, Sarah M Alenezi, Elisa Camela, Andrea D Maderal, Paolo Romanelli

**Affiliations:** 1 Department of Dermatology, Nova Southeastern University College of Allopathic Medicine, Fort Lauderdale, USA; 2 Dr. Phillip Frost Department of Dermatology and Cutaneous Surgery, University of Miami, Miller School of Medicine, Miami, USA; 3 Section of Dermatology, Department of Clinical Medicine and Surgery, University of Naples Federico II, Napoli, ITA

**Keywords:** eccrine adnexal tumor, adnexal neoplasm, adnexal skin tumor, ectopic hidradenoma, hidradenoma papilliferum

## Abstract

Hidradenoma papilliferum (HP) is a benign adnexal tumor, commonly affecting the anogenital region of middle-aged women. Clinically, HP typically presents as a slow-growing, unilateral, well-circumscribed, smooth skin-colored cystic dermal nodule, usually growing less than 1 cm in size. Reports of ectopic HP are exceedingly rare but have been identified in areas containing modified apocrine gland structures, most commonly on the head and neck, and have included ceruminous glands of the external ear canal, the Moll glands of the eyelid, mammary glands of the breast, maxillofacial region and areas on the scalp. To the best of our knowledge, there is only one case of ectopic HP located on the external ear canal reported in English literature. We present a second case of draining ectopic HP located on the conchal bowl of the external ear canal.

## Introduction

Hidradenoma papilliferum (HP) is a benign adnexal tumor, commonly affecting the anogenital region of middle-aged women [1]. HP is thought to proliferate from hormonal stimulation on apocrine glands, as lesions of HP contain estrogen and progesterone receptors [1]. Clinically, HP presents as a slow-growing, unilateral, well-circumscribed, smooth skin-colored cystic dermal papule, typically growing less than 1 cm. Commonly, these lesions are asymptomatic, however, ulcerated HP or malignant transformation of the lesions have been reported [2].

Reports of ectopic HP are exceedingly rare but have been identified in areas containing modified apocrine gland structures, most commonly on the head and neck, and have included ceruminous glands of the external ear canal, the Moll glands of the eyelid, mammary glands of the breast, maxillofacial region and areas on the scalp [2-5]. To the best of our knowledge, there is only one case of ectopic HP located on the external ear canal reported in English literature [3]. We present a second case of draining ectopic HP located on the conchal bowl of the external ear canal.

## Case presentation

**Figure 1:**
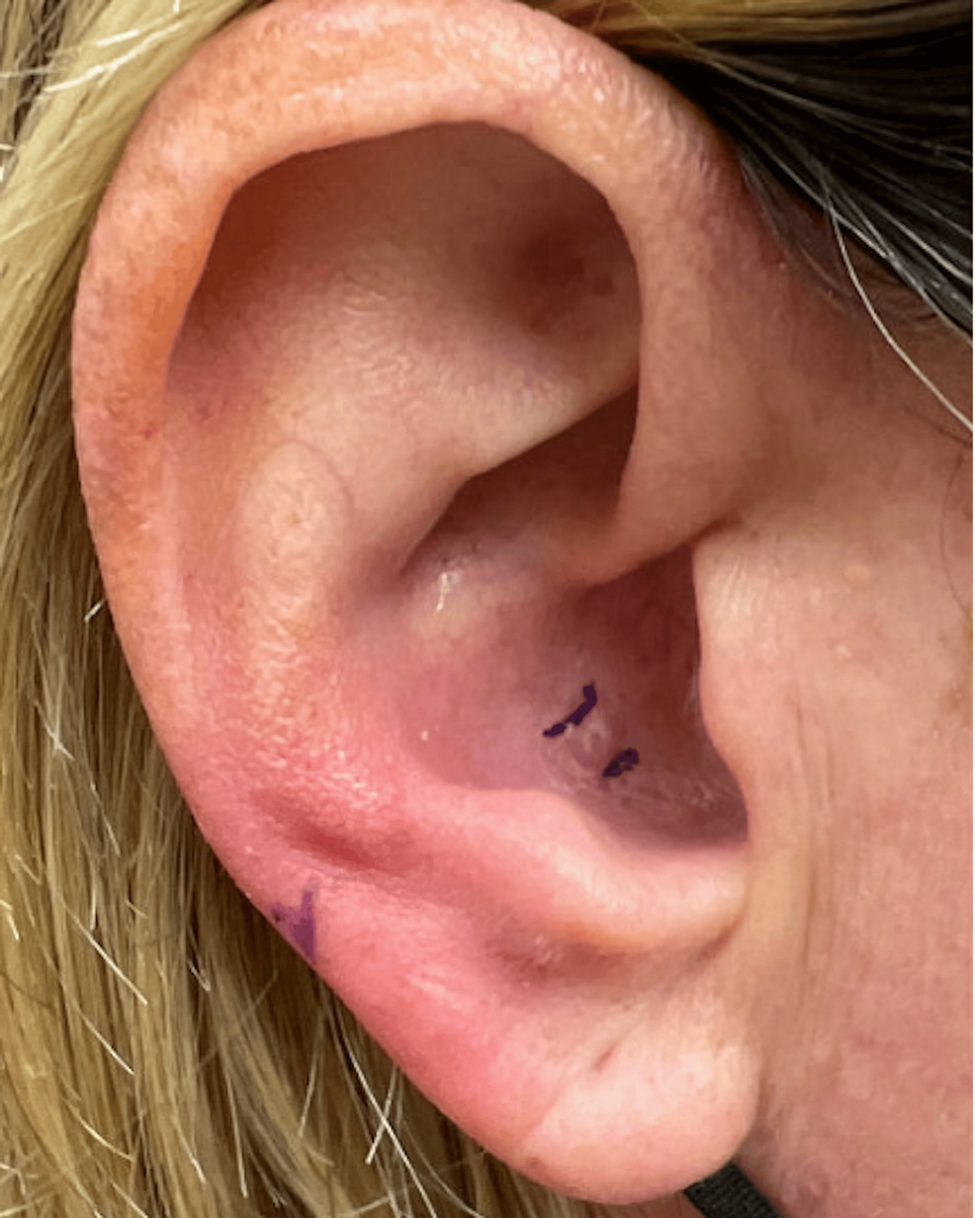
Clinical presentation of patient's skin-colored papule on the conchal bowl of the external auditory canal

A 43-year-old woman presented to our clinic with concerns about a solitary, skin-colored papule located on her right external ear canal that has been present for over 5 years (Figure 1).

**Figure 2:**
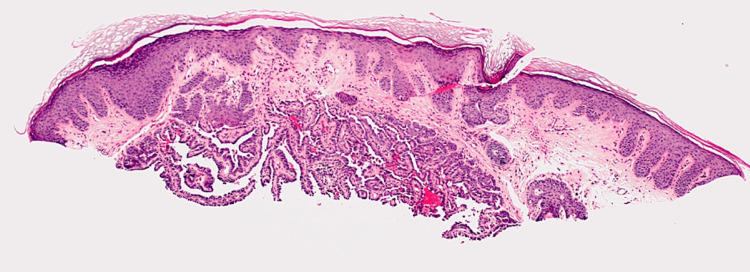
Histologic examination of a shave biopsy performed on the solitary lesion The specimen was paraffin and formalin embedded. Hematoxylin and eosin staining displaying a dermal nodule with papillary features, with no evident connection to the overlying epidermis. Image captured at 4x magnification

The patient reports draining of clear-white fluid from the papule recurrently draining upon self-manipulation. The papule does not decrease in size with the expulsion of the fluid. The patient denies other symptoms of bleeding, ulceration, burning, pain, or itch. She also denies other lesions of similar clinical manifestation. A shave biopsy was performed to rule out cutaneous malignancy, such as basal cell carcinoma. Histological evaluation revealed a dermal nodule with elongated, fibrous stroma in arborizing pattern without connection to the overlying epithelium and no evident plasma cells (Figure 2).

Correlation of the clinical symptomatology with histological examination reveals a diagnosis of HP. Due to its location on the external auditory canal, this HP was considered ectopic in nature, and thus, further excision was performed to reduce the rare risk of cancerous transformation.

## Discussion

The diagnosis of HP is extremely challenging when the location is in atypical sites, typically outside the anogenital region, since the clinical and dermoscopic presentations are unspecific [2]. Moreover, the rarity of the lesion may favor other more prevalent and impactful differential diagnoses, such as basal cell carcinomas, that represent the major indication for surgical excision [5]. Hence, the diagnosis is often incidental following histological evaluation. Common histological findings include the presence of a cystic eosinophilic space, associated with papillary and adenomatous structures, lined with a double layer of epithelial cells. The basal layer contains cuboidal cells, while the luminal layer is made of large columnar cells [6]. The immunohistochemistry (IHC) is positive for epithelial membrane antigen, carcinoembryonic antigen (CEA), gross cystic disease fluid protein‐15, and human milk fat globule membrane antigen, and is often reactive for the markers of apocrine differentiation [2]. In the histological differentials, other entities such as tubular apocrine adenoma, clear cell (apocrine) hidradenoma, and syringocystadenoma papilliferum must be taken into consideration [6]. Traditional surgical excision represents the gold-standard treatment for the management of HP lesions; no further therapies are recommended given the benign character of the lesion. Although reports of malignant transformation have only been observed in typical forms of HP, involving intraductal carcinoma resembling apocrine carcinoma and invasive adenosquamous carcinoma, dedicated follow-up of the excised area is still typically recommended. Interestingly, it has been speculated that some serotypes of human papillomavirus (HPV) may have a role in the malignant evolution of HP, even though such association is yet to be verified [5].

## Conclusions

In conclusion, HP is a benign adnexal tumor commonly located in the anogenital regions of the body. These benign adnexal tumors contain estrogen and progesterone receptors and are thought to proliferate from hormonal stimulation on apocrine glands. Classically, lesions present as solitary skin to pink-colored papules, and may commonly be mistaken for cutaneous malignancies such as basal cell carcinomas. Reports of ectopic locations of HP have been noted, involving areas of eccrine and apocrine activity. Histologic examination typically reveals fibrous stroma with arborizing patterns without connection to the overlying epidermis. Further immunohistochemical staining may be performed and would stain positive for epithelial membrane antigen, CEA, gross cystic disease fluid protein‐15, and human milk fat globule membrane antigen, and is often reactive for the markers of apocrine differentiation. Close follow-up of surgically excised lesions is encouraged due to the exceedingly rare, but prevalent, risk of malignant transformation.
